# Bayesian Spatio-Temporal Modeling of the Dynamics of COVID-19 Deaths in Peru

**DOI:** 10.3390/e26060474

**Published:** 2024-05-30

**Authors:** César Raúl Castro Galarza, Omar Nolberto Díaz Sánchez, Jonatha Sousa Pimentel, Rodrigo Bulhões, Javier Linkolk López-Gonzales, Paulo Canas Rodrigues

**Affiliations:** 1 Escuela de Posgrado, Universidad Peruana Unión, Lima 15468, Peru; cesarcastrogalarza@gmail.com (C.R.C.G.); omarndisa@gmail.com (O.N.D.S.); javierlinkolk@gmail.com (J.L.L.-G.); 2 Department of Statistics, Federal University of Pernambuco, Recife 50740-540, PE, Brazil; 3 Department of Statistics, Federal University of Bahia, Salvador 40170-110, BA, Brazil; rbulhoes@ufba.br (R.B.); paulocanas@gmail.com (P.C.R.)

**Keywords:** COVID-19, spatio-temporal modeling, areal unit data, spatio-temporal generalized linear model, Bayesian statistics

## Abstract

Amid the COVID-19 pandemic, understanding the spatial and temporal dynamics of the disease is crucial for effective public health interventions. This study aims to analyze COVID-19 data in Peru using a Bayesian spatio-temporal generalized linear model to elucidate mortality patterns and assess the impact of vaccination efforts. Leveraging data from 194 provinces over 651 days, our analysis reveals heterogeneous spatial and temporal patterns in COVID-19 mortality rates. Higher vaccination coverage is associated with reduced mortality rates, emphasizing the importance of vaccination in mitigating the pandemic’s impact. The findings underscore the value of spatio-temporal data analysis in understanding disease dynamics and guiding targeted public health interventions.

## 1. Introduction

The emergence of Coronavirus Disease 2019 (COVID-19) in December 2019 marked the beginning of a global crisis, with the World Health Organization (WHO) declaring it a pandemic in March 2020 due to its alarming severity and rapid spread. COVID-19, caused by the SARS-CoV-2 virus, belongs to the family of coronaviruses and has since posed significant health, social, and economic challenges worldwide [1,2,3].

Peru has faced immense challenges in combating the COVID-19 pandemic, like many other countries. The first case of COVID-19 was confirmed on 6 March 2021, triggering a rapid escalation in cases and subsequent waves of infection. The pandemic has had significant impacts in Peru, including economic challenges, disruptions to education, and mental health concerns, leaving many children orphaned and highlighting vulnerabilities in healthcare systems [4,5]. Peru was one of the worst-affected countries in cases and deaths [6].

To combat the pandemic, scientific collaborations expedited the development and authorization of COVID-19 vaccines, which have become pivotal in preventing the spread of the disease and reducing mortality rates [7,8]. However, achieving widespread vaccination coverage remains challenging, particularly in regions with limited healthcare resources [9].

In this context, spatio-temporal data analysis plays a crucial role in understanding the dynamics of the pandemic, identifying high-risk areas, and informing public health interventions for similar future outbreaks. Despite its importance, few studies have been conducted to help understand the spatial and temporal trends of COVID-19 in Peru. An exception is the study of [10], which demonstrated the feasibility and value of implementing a decentralized SARS-CoV-2 RNA wastewater monitoring system to assess the spatio-temporal distribution of COVID-19 in three major cities in Peru. On the other hand, several studies have proposed using spatio-temporal approaches to model the number of deaths and the number of cases of COVID-19 data as outlined by a review that summarizes the contribution of spatio-temporal modeling methodologies in the field of COVID-19 [11]. For example, [12] proposed a spatio-temporal modeling approach of COVID-19 incident cases in Italy, ref. [13] used a model based on discrete latent variables, which are spatially associated and time-specific, for the analysis of incident cases in Italy, ref. [14] studied the association between COVID-19 spread and environmental conditions in Catalonia, Spain, ref. [15] studied the association between deaths and impact of key risk factors in England, ref. [16] studied risk clusters of COVID-19 transmission in northeastern Brazil, ref. [17] analyzed the behavior and forecast of the number of deaths in the 26 Brazilian states, the federal district, and Italy, and [18] presented a spatio-temporal modeling analysis of initial COVID-19 diffusion in China and in the United States.

Despite the wealth of spatio-temporal COVID data available, there remains a gap in the literature regarding applying advanced statistical spatio-temporal models to analyze and understand the dynamics of these datasets, particularly for Peru. This work aims to fill this gap in two ways. Firstly, it provides a descriptive and exploratory analysis of the spatial and temporal dynamics of COVID-19 data in Peru. Then, we employ a spatio-temporal generalized linear model tailored for areal unit data, with parameter inferences conducted within a Bayesian framework. By leveraging this model, we aim to elucidate the spatial and temporal patterns of COVID-19 deaths, investigate the impact of vaccination efforts, and provide valuable insights for guiding public health policies and interventions.

This paper is structured as follows. Section 2 describes the data sources and preprocessing steps and outlines the methodology, including the Bayesian spatio-temporal generalized linear model and Bayesian inference framework. Section 3 presents the results and discussion of the analysis, including the descriptive and exploratory analysis of the temporal and spatial dynamics of the data and the results of the statistical model. Finally, Section 4 concludes with a summary of key findings and suggestions for future research.

## 2. Materials and Methods

This section provides an overview of the data utilized in our analysis, including the collection, processing, and organization procedures of both the response variable and the covariates. We then detail the construction of the spatio-temporal generalized linear model tailored for areal unit data, whose parameter inferences are performed within a Bayesian framework.

### 2.1. The Data

This study examines data spanning two years, from 3 March 2020 to 13 December 2021. The data include information from three distinct sources:

(i) Records of COVID-19 deaths across Peru sourced from the Ministerio de Salud (MINSA) and accessible through the Plataforma Nacional De Datos Abiertos (www.datosabiertos.gob.pe/);
(ii) Records of COVID-19 cases across Peru sourced from the MINSA;
(iii) Vaccination records against COVID-19, also obtained from the MINSA.

**Figure 1:**
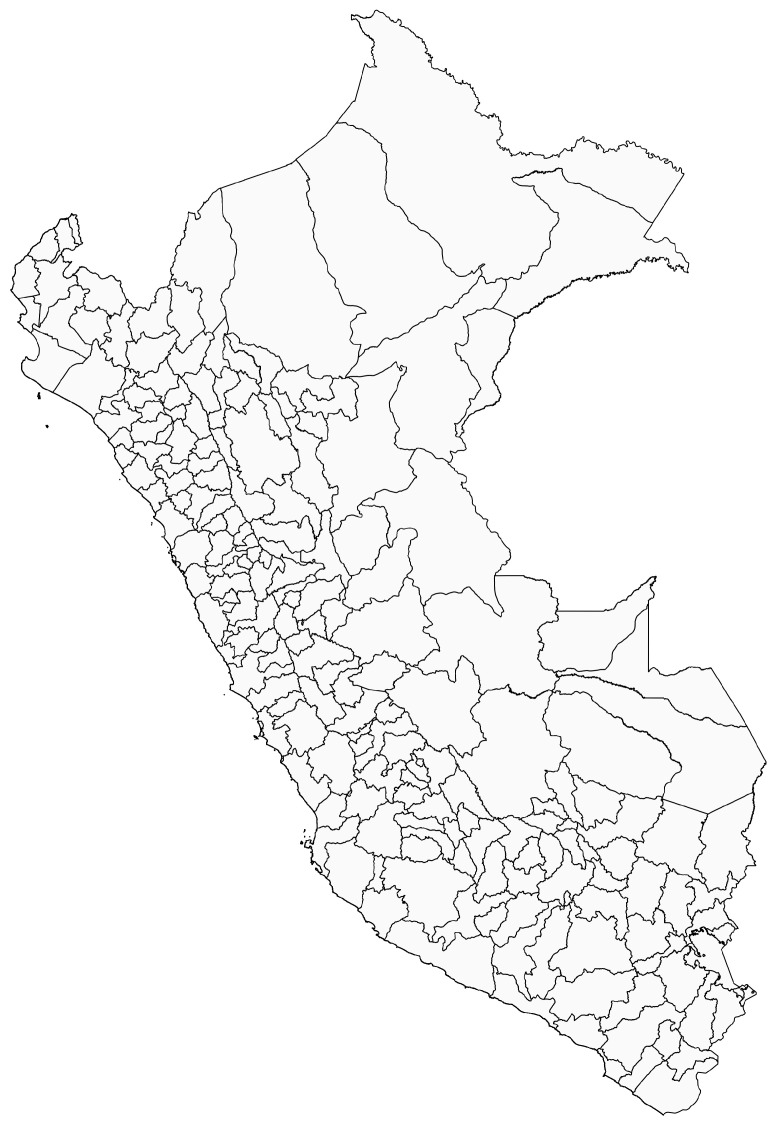
Map of Peru subdivided by its 194 provinces and Lake Titicaca.

Peru is geographically divided into three levels: 26 regions, 194 provinces, and 1838 districts. Furthermore, the shapefile includes Lake Titicaca because of its extension. It is located on the border between Peru and Bolivia in the Andes Mountains and is one of the largest lakes in South America. Therefore, this part of the territory will not be included in the analysis despite being represented in the figures. For the modeling phase, the data were aggregated at the province level to provide spatial coverage across the country. This division is illustrated in Figure 1. Temporally, the data were organized daily, resulting in a panel format dataset. Each column represents one of the 194 Peruvian provinces, and each row represents a date, resulting in 651 observations for each province. The cutoff date for the analysis was set as 13 December 2021, corresponding to the date of the last death recorded in the dataset of deceased individuals by COVID-19.

Given the diverse formats for the datasets, several approaches were employed to transform them into the final format required for analysis. Detailed descriptions of data collection, processing, and organization are provided below to ensure transparency and facilitate reproducibility in scientific research.

The response variable, the number of deaths from COVID-19, initially resided in a single file encompassing all recorded deaths within Peruvian territory from 3 March 2020 to 13 December 2021. Over this period, slightly over 200,000 deaths from COVID-19 were documented, corresponding to the total number of entries in the dataset. Deaths were aggregated based on the date and province of occurrence to derive the final dataset, resulting in a structured dataset with 651 rows and 194 columns. Each row represents a day within the analysis period, while each column represents a Peruvian province. We assume that a zero value was assigned for days where no observations were recorded in a particular province, meaning the absence of reported deaths during that specific day in that location.

The variable total COVID-19 cases was initially consolidated into a single file containing all cases registered across the Peruvian territory between 6 March 2020 and 31 December 2023. However, due to the absence of response variable data for a period equivalent to the variables, the analysis period was restricted to 3 March 2020 to 13 December 2021. Thus, the three days of difference between the beginning of recording cases and deaths were assumed as zero cases. This raw file comprised over ten variables, including information about people, such as age, sex, geographic information, and district and province of case notification. Throughout this period, slightly over 4.5 million COVID-19 cases were documented in Peru, corresponding to the total number of entries in the database. Subsequently, reported cases were aggregated based on the notification date and province of residence for model development and application. This process yielded a dataset with dimensions of 651 rows and 194 columns, where each row represents a day within the analysis period and each column represents a Peruvian province. Similarly to the number of deaths, we assume that a zero value was assigned for days where no observations were recorded in a given province, meaning the absence of reported cases during that specific day in that location.

Due to its considerable size, the COVID-19 vaccination database posed significant challenges in reading and processing. The original dataset comprises a single file containing all records of COVID-19 vaccines administered in Peru from 27 April 2020 to 29 November 2023, totaling over 90 million doses administered nationwide, corresponding to the total number of rows in the database. Similarly to the previous variables, the data are limited between 3 March 2020 and 13 December 2021. The raw file contains 14 variables, including the vaccination location, the vaccinated individual’s risk group (whether the person who was vaccinated belonged to a risk group for worsening the disease), gender, and vaccine manufacturer (i.e., the company responsible for the vaccine). The number of vaccines administered was aggregated based on the location and day of administration. Additionally, the data were segmented into four distinct subsets corresponding to the first four doses administered, each comprising 651 rows (representing the number of days) and 194 columns (representing provinces).

The dataset used for the analysis and modeling consists, for each Peruvian province, of the number of deaths from COVID-19, the response variable in this study, and the explanatory variables total number of COVID-19 cases and number of vaccine doses against COVID-19 administered, divided into four categories corresponding to the first four doses. The response variable and the five covariates were transformed into seven-day moving averages to correct for large weekly fluctuations. Furthermore, a difference of 14 days was adopted between the response variable number of deaths and the explanatory variables, which can be justified by the incubation time and presentation of symptoms that varied between 2 and 14 days [19,20,21].

All analyses were performed using the R programming language (version 4.3.2) [22] on a personal computer equipped with a 2.50 GHz Intel Core i5-10300H processor and 8 GB of RAM, running on the Windows 11 64-bit operating system.

### 2.2. Bayesian Spatio-Temporal Model

The conventional linear regression model typically assumes independent observations and overlooks temporal and spatial dependencies. However, considering the nature of our panel data, which varies across time and space, it becomes imperative to account for these dependencies. To address this challenge, we adopted a Bayesian spatio-temporal generalized linear model specifically tailored for areal unit data, which was proposed by [23] and whose main ideas were previously developed by [24,25]. This model is well suited for fitting areal unit data observed in discrete periods while including relevant explanatory variables. Next, we will briefly review the formulation of this model that we will apply to analyze our data.

Let $Y_{k,t}$ be the response variable in areal unit *k* and time *t*, where k∈{1,…,K}, t∈{1,…,N}, *K* is the total of areal units, and *N* is the number of periods of time. Suppose that $Y_{k,t}|\mu_{k,t}∼\mathrm{Poisson}\left(E_{k,t}\mu_{k,t}\right)$ and $\ln\mu_{k,t}=x_{k,t}^{⊺}\beta +O_{k,t}+\psi_{k,t}$, where $\mu_{k,t}$ is the risk of death in areal unit *k* and time *t* relative to the expected counts $E_{k,t}$, $x_{k,t}$ is a *p*-variate vector of known covariates for areal unit *k* and time *t*, $\beta =(\beta_{1},\ldots ,\beta_{p})$ is a *p*-dimensional vector of covariate regression parameters, and $O_{k,t}$ is an offset for areal unit *k* and time *t*. Including a random effects term $\psi_{k,t}$ for areal unit *k* and time *t* allows us to effectively capture the inherent dependencies of our dataset that vary over time and space. Other model specifications are given by

(1) $$\begin{array}{ccc} \beta & ∼ & \mathrm{Normal}(\mu_{\beta },\Sigma_{\beta }) \end{array}$$

(2) $$\begin{array}{ccc} \psi_{k,t} & = & \left(\beta_{1}+\phi_{k}\right)+\left(\alpha +\delta_{k}\right)\frac{t-\bar{t}}{N} \end{array}$$

(3) $$\begin{array}{ccc} \phi_{k}|\phi_{-k},W & ∼ & \mathrm{Normal}\left(\mu_{\phi_{k}|\phi_{-k},W},\sigma_{\phi_{k}|\phi_{-k},W}^{2}\right) \end{array}$$

(4) $$\begin{array}{ccc} \delta_{k}|\delta_{-k},W & ∼ & \mathrm{Normal}\left(\mu_{\delta_{k}|\delta_{-k},W},\sigma_{\delta_{k}|\delta_{-k},W}^{2}\right) \end{array}$$

(5) $$\begin{array}{ccc} \alpha & ∼ & \mathrm{Normal}(\mu_{\alpha },\sigma_{\alpha }^{2}), \end{array}$$

where

- $\bar{t}=\sum_{t=1}^{N}t/N$ is the average time;
- $\psi_{k,t}$ is a latent component for the province *k* and day *t* embracing one or more sets of spatio-temporally autocorrelated random effects, which are known as *correlated linear time trends* [26];
- $W=[w_{k,j}]$ is a binary neighborhood matrix K×K, with $w_{k,j}=0$ if k=j (diagonal elements equal to zero), $w_{k,j}=1$ if the provinces *k* and *j* share a common border, and $w_{k,j}=0$ if the provinces *k* and *j* do not share a common border;
- The random effects $\phi =(\phi_{1},\ldots ,\phi_{K})$ and $\delta =(\delta_{1},\ldots ,\delta_{K})$ are modeled as spatially autocorrelated by the CAR (conditional autoregressive) prior, satisfying $\sum_{k=1}^{K}\phi_{k}=\sum_{k=1}^{K}\delta_{k}=0$ to avoid a lack of identifiability, with $\phi_{-k}$ and $\delta_{-k}$ denoting the vectors ϕ and δ without their corresponding *k*th entries, respectively;
- The quantities $\mu_{\phi_{k}|\phi_{-k},W}$, $\sigma_{\phi_{k}|\phi_{-k},W}^{2}$, $\mu_{\delta_{k}|\delta_{-k},W}$, and $\sigma_{\delta_{k}|\delta_{-k},W}^{2}$ are given, respectively, by
  (6) $$\mu_{\phi_{k}|\phi_{-k},W}=\frac{\rho_{int}\sum_{j=1}^{K}w_{k,j}\phi_{j}}{\rho_{int}\sum_{j=1}^{K}w_{k,j}+1-\rho_{int}},$$
  (7) $$\sigma_{\phi_{k}|\phi_{-k},W}^{2}=\frac{\tau_{int}^{2}}{\rho_{int}\sum_{j=1}^{K}w_{k,j}+1-\rho_{int}},$$
  (8) $$\mu_{\delta_{k}|\delta_{-k},W}=\frac{\rho_{slo}\sum_{j=1}^{K}w_{k,j}\delta_{j}}{\rho_{slo}\sum_{j=1}^{K}w_{k,j}+1-\rho_{slo}},$$
  and
  (9) $$\sigma_{\delta_{k}|\delta_{-k},W}^{2}=\frac{\tau_{slo}^{2}}{\rho_{slo}\sum_{j=1}^{K}w_{k,j}+1-\rho_{slo}}.$$

The model captures the spatio-temporal pattern in the mean response by incorporating a spatially varying linear time trend. Each province (indexed by *k*) possesses its own individual linear time trend, characterized by a spatially varying intercept $\beta_{1}+\phi_{k}$ and a spatially varying slope $\alpha +\delta_{k}$.

The parameters $\rho_{int}$ and $\rho_{slo}$ are spatial dependence parameters, with zero corresponding to independence and one corresponding to strong spatial smoothness. Each parameter follows a uniformly distributed prior distribution over the unit interval. The random effects variances are specified with the conjugate prior distributions $\tau_{int}^{2}∼\mathrm{Inverse}\text{-}\mathrm{Gamma}\left(a,b\right)$ and $\tau_{slo}^{2}∼\mathrm{Inverse}\text{-}\mathrm{Gamma}\left(a,b\right)$.

To have flat prior distributions for all model parameters, we specify the following hyperparameters: $\mu_{\beta }=0_{p}$, $\Sigma_{\beta }=10^{5}\cdot I_{p}$, a=0, b=0.01, $\mu_{\alpha }=0$, and $\sigma_{\alpha }^{2}=1000$. This statistical model is implemented in the function *ST .CARlinear* of the R package CARBayesST [26], which estimates the model parameters using the MCMC (Markov chain Monte Carlo) algorithm.

The spatio-temporal modeling aims to characterize the total number of COVID-19 deaths in Peru over a specified period, aggregated by provinces and days. Our analysis incorporates explanatory variables such as the total number of COVID-19 cases and the cumulative count of the first four doses of the COVID-19 vaccine administered in the respective provinces.

## 3. Results and Discussion

### 3.1. Descriptive and Exploratory Analysis

**Figure 2:**
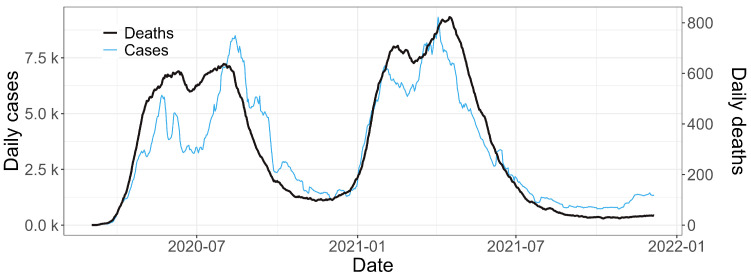
Seven-day moving average for the total COVID-19 cases and deaths in Peru between 3 March 2020 and 13 December 2021. The 14-day delay used in the modeling phase is not represented in this image.

Our study aims to compare the dynamics of COVID-19 deaths in Peru over a span of two years, examining the impacts of vaccination efforts and the spatial distribution of the disease on mortality. When analyzing the relationship between the number of COVID-19 cases and deaths during the study period spanning from 3 March 2020 to 13 December 2021, as depicted in Figure 2, we observed spikes in cases and deaths in periods of similar behavior between the two series. Specifically, increases in COVID-19 cases correspond to rises in deaths, indicating a consistent pattern across the two variables.

**Figure 3:**
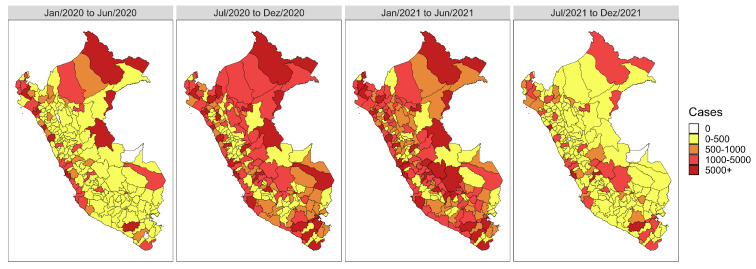
Heatmaps with the evolution in the total number of COVID-19 cases in Peru semiannually between 3 March 2020 and 13 December 2021.

**Figure 4:**
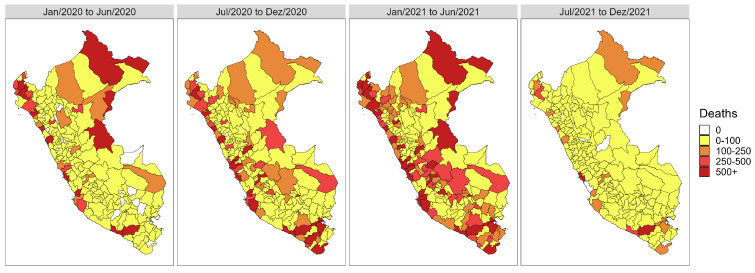
Heat maps with the evolution in the total number of COVID-19 deaths in Peru semiannually between 3 March 2020 and 13 December 2021.

The peaks in COVID-19 cases and deaths are also evident in Figure 3 and Figure 4, which display the cumulative cases and deaths over six-month periods. Specifically, we observe a higher concentration of cases during the second half of 2020 and the first half of 2021, corresponding to the periods where the highest peaks occurred in the series (Figure 2). Similarly, the number of deaths also exhibits distinct periods, with notable variations across the three periods encompassing 2020 and the first half of 2021.

**Figure A1:**
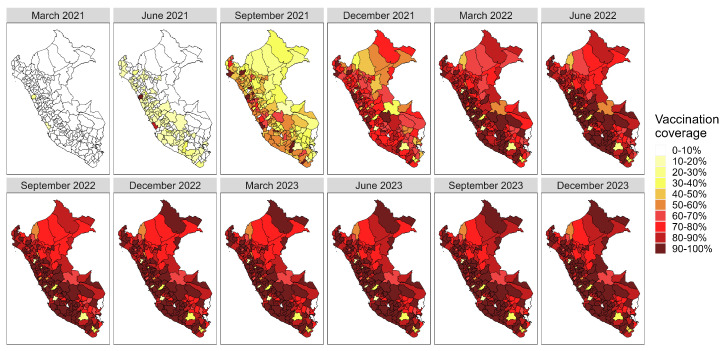
Heatmaps with the vaccine coverage of the first COVID-19 dose every three months during the COVID-19 pandemic across all 194 provinces of Peru and Lake Titicaca.

**Figure A2:**
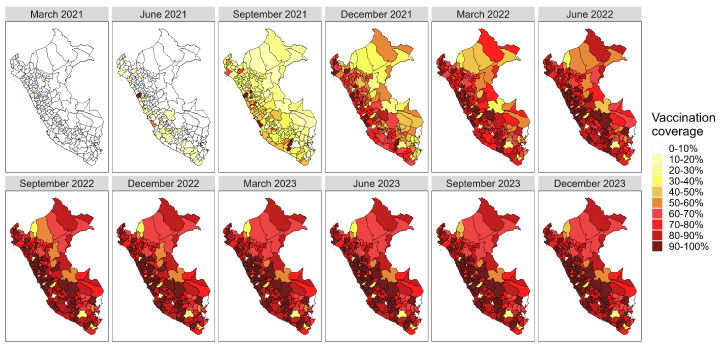
Heat maps with the vaccine coverage of the second COVID-19 dose every three months during the COVID-19 pandemic across all 194 provinces of Peru and Lake Titicaca.

**Figure A3:**
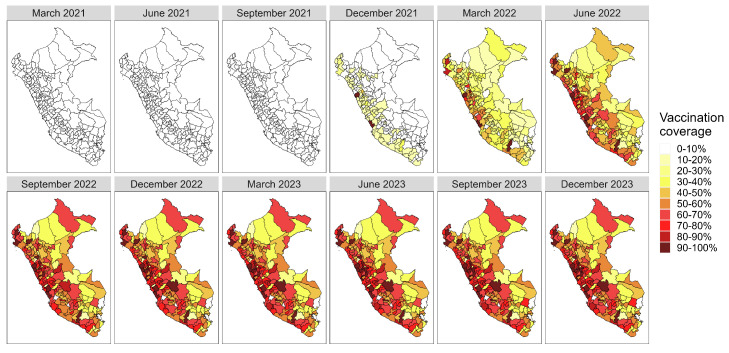
Heat maps with the vaccine coverage of the third COVID-19 dose every three months during the COVID-19 pandemic across all 194 provinces of Peru and Lake Titicaca.

**Figure A4:**
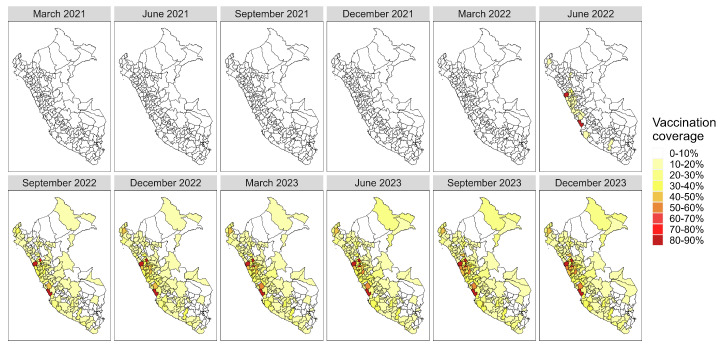
Heat maps with the vaccine coverage of the fourth COVID-19 dose every three months during the COVID-19 pandemic across all 194 provinces of Peru and Lake Titicaca.

Figure A1, Figure A2, Figure A3 and Figure A4 of Appendix A show the heat maps with the vaccine coverage of the first four COVID-19 doses, respectively, every three months during the COVID-19 pandemic across all 194 provinces of Peru and Lake Titicaca.

### 3.2. Bayesian Spatio-Temporal Modeling

After the initial descriptive and exploratory analysis, it became apparent that the (seven-day moving average) number of deaths exhibits spatial and temporal dependencies. Acknowledging this dependence, a spatio-temporal model was estimated for the entire period. In this context, we utilized the Bayesian spatio-temporal generalized linear model proposed by [23], chosen for its suitability in handling count data and enabling the incorporation of explanatory variables. The percentage of the population per province has been included as an offset for our model to account for the population difference between provinces.

We analyzed data from K=194 provinces over a period of N=651 days. We assessed the correlation strength between each candidate variable and the response variable in each model to select explanatory variables for each model, and we used different combinations of models with the available variables, selecting the one in which the variables were jointly statistically significant, in a backward manner. We ran the MCMC algorithm 20,000 times to estimate the model parameters, excluding the first 2000 iterations (warm-up period) and taking samples from the posterior distribution every 6th iteration to reduce autocorrelation in the chains (thinning).

During the estimation process, two models (with and without a 14-day lag between the number of cases and other variables) were compared based on the deviance information criterion (DIC), the log marginal predictive likelihood (LMPL), and the Watanabe–Akaike information criterion (WAIC). The model that considered a 14-day lag between the number of cases and other variables showed an improvement of about 10% in the evaluation metrics DIC, WAIC, and LMPL. Table 1 presents the most relevant coefficients of the model, including the degree of spatial dependence and the significance of the coefficients associated with the covariates.

Under the Bayesian framework employed in this study, we consider each component of β to be statistically significant when the value of zero falls outside the credibility interval formed by the 2.5th and 97.5th percentiles of its corresponding posterior distribution. Among the covariates considered, it was observed that the (seven-day moving average) number of cases and the second and third doses of the COVID-19 vaccine were statistically significant and kept in the model. Moreover, based on the coefficients, it is evident that the (seven-day moving average) number of cases positively influences the number of deaths. At the same time, both doses of the vaccine exert a negative influence, with the third dose showing a more pronounced reduction effect.

The term $\delta_{k}$ is known as the “differential trend of the *k*th areal unit”, which is the interaction between the time effect and the area effect. An interpretation for the time effect is provided by [24] as follows: a value of $\delta_{k}<0$ implies that the temporal trend of the areal unit *k* is less steep than the mean trend α, while a value of $\delta_{k}>0$ implies that the temporal trend of areal unit *k* is steeper than the mean trend α.

**Figure 5:**
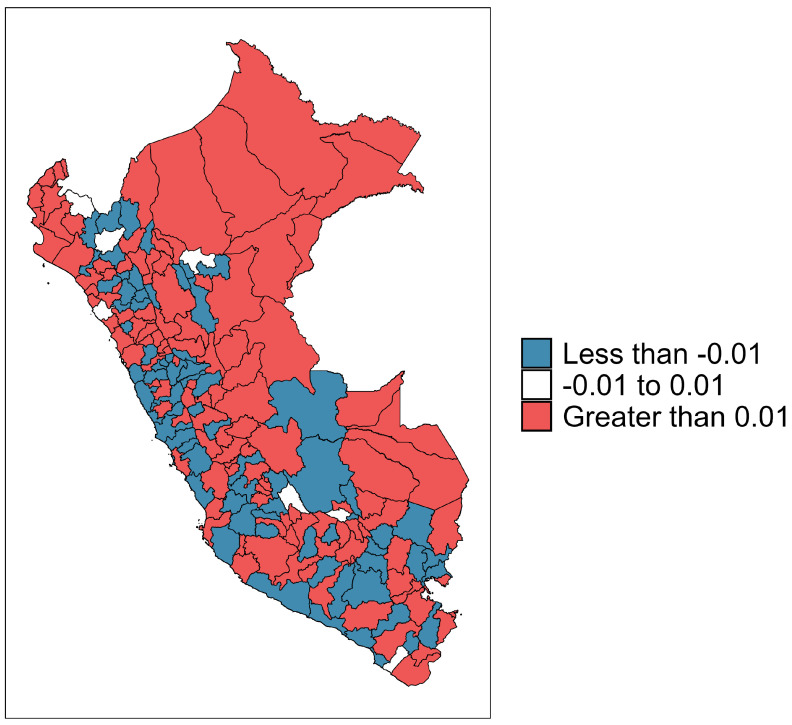
Heatmaps with the posterior mean of the parameter $\delta_{k}$ of the spatio-temporal model across all provinces of Peru, where *k* indicates the *k*th province.

Figure 5 depicts the posterior average of the parameter $\delta_{k}$, where *k* denotes the *k*th province. The areal units whose trend is steeper than the average trend are further east and north. From this map, it is evident that a diverse array of behaviors and temporal trends are present. Provinces closer to the capital and along the coast exhibit negative temporal trends more frequently. Conversely, provinces located farther from the capital and in the country’s northern regions display positive temporal trends, indicating an upward trend in the (seven-day moving average) number of deaths over the study period.

**Figure A5:**
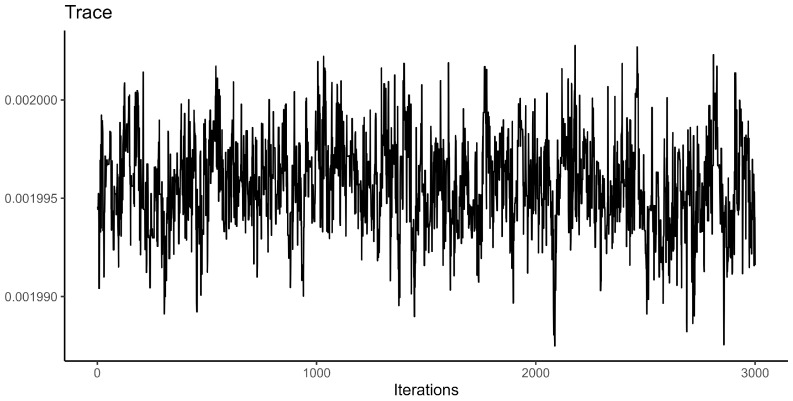
Trace plots for model parameter $\beta_{Cases}$.

Based on Table 1, the MCMC algorithm achieves convergence. When the Geweke statistic takes high values, say, outside the interval (−1.96,1.96), it indicates convergence problems [27], which is not observed for any of the model parameters. Although acceptance rates for certain parameters were low, visual diagnosis based on trace plots (Figure A5 shows the trace plot related to $\beta_{Cases}$) also shows convergence.

## 4. Concluding Remarks

In conclusion, our study sheds light on the complex spatial and temporal dynamics of COVID-19 in Peru and underscores the critical role of vaccination in mitigating mortality rates. The findings highlight the effectiveness of vaccination campaigns in reducing the impact of the pandemic and emphasize the importance of continued efforts to enhance vaccine coverage. Furthermore, our analysis underscores the value of spatio-temporal data analysis in guiding targeted public health interventions and the importance of effective case management and prevention strategies. Moving forward, policymakers and healthcare authorities should prioritize vaccination efforts and implement evidence-based interventions to control the spread of COVID-19 and minimize its impact on communities. By leveraging advanced statistical models and comprehensive data analysis, we can better understand disease dynamics and inform strategies to combat future outbreaks effectively. Overall, this study contributes valuable insights to the global effort to address the COVID-19 pandemic and provides a foundation for further research in this critical area of public health.

For future research, it is essential to continue monitoring the spatial and temporal dynamics of COVID-19, particularly in the context of emerging variants and changing vaccination strategies. Longitudinal studies are needed to assess the long-term effectiveness of vaccination campaigns and the persistence of immunity over time. Furthermore, research on the socio-economic and demographic determinants of COVID-19 mortality could provide valuable insights into disparities in disease burden and inform targeted interventions to address health inequities. Additionally, incorporating real-time data and advanced modeling techniques could enhance our ability to effectively predict and respond to future outbreaks. Moreover, exploring the application of spatio-temporal modeling techniques to other fields of study, such as infectious disease epidemiology [28], environmental health [29,30,31], natural disaster management [32], and climate change [33,34,35], could provide further insights into complex systems’ dynamics and inform evidence-based decision making across various domains. Overall, continued research in this area is crucial for informing evidence-based strategies to control the spread of COVID-19 and other infectious diseases and minimize their impact on public health.

## Figures and Tables

**Table 1:** Posterior mean, 2.5th, and 97.5th percentiles of the posterior distribution of the model parameters. The acceptance percentage, effective sample size, and Geweke statistic for each model parameter are provided. The sample size of the posterior distribution of each parameter is equal to one thousand. DIC: 621,729; WAIC: 640,586; LMPL: −316,548.

Parameter	Posterior	2.5th Perc.	97.5th Perc.	% Accept.	Effective	Geweke
	Mean				Samples	
Intercept	0.0722	0.0657	0.0789	13	128	−0.3
$\beta_{Cases}$	0.0020	0.0020	0.0020	13	285	0.4
$\beta_{Vaccine2}$	−0.1354	−0.1462	−0.1243	13	105	−0.1
$\beta_{Vaccine3}$	−0.4078	−0.4334	−0.3808	13	105	−0.1
α	−0.1337	−0.1535	−0.1148	38	2263	−1.4
$\tau_{int}^{2}$	0.0888	0.0675	0.1228	100	1610	−0.2
$\tau_{slo}^{2}$	0.1506	0.1124	0.2063	100	1907	0.1
$\rho_{int}$	0.0335	0.0010	0.1171	41.6	1523	0.1
$\rho_{slo}$	0.0287	0.0008	0.1003	43.2	1524	−0.2

## Data Availability

The data used in this paper are available at https://github.com/SaLLy-laboratory/COVID19-Peru (accessed on 27 May 2024).

## References

[1] Baloch S., Baloch M.A., Zheng T., Pei X. (2020). The coronavirus disease 2019 (COVID-19) pandemic. Tohoku J. Exp. Med..

[2] Ciotti M., Ciccozzi M., Terrinoni A., Jiang W.C., Wang C.B., Bernardini S. (2020). The COVID-19 pandemic. Crit. Rev. Clin. Lab. Sci..

[3] Abenavoli L., Gentile I. (2023). COVID-19: Where We Are and Where We Are Going. Diseases.

[4] Alvarez-Risco A., Mejia C.R., Delgado-Zegarra J., Del-Aguila-Arcentales S., Arce-Esquivel A.A., Valladares-Garrido M.J., Del Portal M.R., Villegas L.F., Curioso W.H., Sekar M.C. (2020). The Peru approach against the COVID-19 infodemic: Insights and strategies. Am. J. Trop. Med. Hyg..

[5] Schwalb A., Seas C. (2021). The COVID-19 pandemic in Peru: What went wrong?. Am. J. Trop. Med. Hyg..

[6] Karlinsky A., Kobak D. (2021). Tracking excess mortality across countries during the COVID-19 pandemic with the World Mortality Dataset. eLife.

[7] Ndwandwe D., Wiysonge C.S. (2021). COVID-19 vaccines. Curr. Opin. Immunol..

[8] Lopez Bernal J., Andrews N., Gower C., Gallagher E., Simmons R., Thelwall S., Stowe J., Tessier E., Groves N., Dabrera G. (2021). Effectiveness of COVID-19 vaccines against the B. 1.617. 2 (Delta) variant. N. Engl. J. Med..

[9] Calina D., Docea A.O., Petrakis D., Egorov A.M., Ishmukhametov A.A., Gabibov A.G., Shtilman M.I., Kostoff R., Carvalho F., Vinceti M. (2020). Towards effective COVID-19 vaccines: Updates, perspectives and challenges. Int. J. Mol. Med..

[10] Pardo-Figueroa B., Mindreau-Ganoza E., Reyes-Calderon A., Yufra S.P., Solorzano-Ortiz I.M., Donayre-Torres A.J., Antonini C., Renom J.M., Quispe A.M., Mota C.R. (2022). Spatiotemporal surveillance of SARS-CoV-2 in the sewage of three major urban areas in Peru: Generating valuable data where clinical testing is extremely limited. Acs Es T Water.

[11] Wang P., Zheng X., Liu H. (2022). Simulation and forecasting models of COVID-19 taking into account spatio-temporal dynamic characteristics: A review. Front. Public Health.

[12] Mingione M., Di Loro P.A., Farcomeni A., Divino F., Lovison G., Maruotti A., Lasinio G.J. (2022). Spatio-temporal modelling of COVID-19 incident cases using Richards’ curve: An application to the Italian regions. Spat. Stat..

[13] Bartolucci F., Farcomeni A. (2022). A spatio-temporal model based on discrete latent variables for the analysis of COVID-19 incidence. Spat. Stat..

[14] Briz-Redón Á. (2021). The impact of modelling choices on modelling outcomes: A spatio-temporal study of the association between COVID-19 spread and environmental conditions in Catalonia (Spain). Stoch. Environ. Res. Risk Assess..

[15] Sartorius B., Lawson A., Pullan R. (2021). Modelling and predicting the spatio-temporal spread of COVID-19, associated deaths and impact of key risk factors in England. Sci. Rep..

[16] Gomes D.S., Andrade L.A., Ribeiro C.J.N., Peixoto M., Lima S., Duque A., Cirilo T.M., Góes M., Lima A., Santos M. (2020). Risk clusters of COVID-19 transmission in northeastern Brazil: Prospective space–time modelling. Epidemiol. Infect..

[17] Pereira C.A.d.B., Nakamura L.R., Rodrigues P.C. (2021). Naive statistical analyses for COVID-19: Application to data from Brazil and Italy. Rev. Bras. Biom..

[18] Griffith D., Li B. (2021). Spatial-temporal modeling of initial COVID-19 diffusion: The cases of the Chinese Mainland and Conterminous United States. Geo-Spat. Inf. Sci..

[19] Lauer S.A., Grantz K.H., Bi Q., Jones F.K., Zheng Q., Meredith H.R., Azman A.S., Reich N.G., Lessler J. (2020). The incubation period of coronavirus disease 2019 (COVID-19) from publicly reported confirmed cases: Estimation and application. Ann. Intern. Med..

[20] Ejima K., Kim K.S., Ludema C., Bento A.I., Iwanami S., Fujita Y., Ohashi H., Koizumi Y., Watashi K., Aihara K. (2021). Estimation of the incubation period of COVID-19 using viral load data. Epidemics.

[21] Men K., Li Y., Wang X., Zhang G., Hu J., Gao Y., Han A., Liu W., Han H. (2023). Estimate the incubation period of coronavirus 2019 (COVID-19). Comput. Biol. Med..

[22] R Core Team (2023). R: A Language and Environment for Statistical Computing.

[23] Lee D., Rushworth A., Napier G. (2018). Spatio-temporal areal unit modeling in R with conditional autoregressive priors using the CARBayesST package. J. Stat. Softw..

[24] Bernardinelli L., Clayton D., Pascutto C., Montomoli C., Ghislandi M., Songini M. (1995). Bayesian analysis of space—Time variation in disease risk. Stat. Med..

[25] Leroux B., Lei X., Breslow N., Bernado J.M., Berger J.O., Dawid A.P., Smith A.F.M. (2000). Estimation of Disease Rates in Small Areas: A new Mixed Model for Spatial Dependence. Statistical Models in Epidemiology, the Environment, and Clinical Trials.

[26] Lee D. (2020). A tutorial on spatio-temporal disease risk modelling in R using Markov chain Monte Carlo simulation and the CARBayesST package. Spat. Spatio-Temporal Epidemiol..

[27] Geweke J., Bernado J.M., Berger J.O., Dawid A.P., Smith A.F.M. (1992). Evaluating the Accuracy of Sampling-Based Approaches to the Calculation of Posterior Moments. Bayesian Statistics.

[28] Ganesan S., Subramani D. (2021). Spatio-temporal predictive modeling framework for infectious disease spread. Sci. Rep..

[29] López-Gonzales J.L., Gómez Lamus A.M., Torres R., Canas Rodrigues P., Salas R. (2023). Self-Organizing Topological Multilayer Perceptron: A Hybrid Method to Improve the Forecasting of Extreme Pollution Values. Stats.

[30] Solci C.C., Reisen V.A., Rodrigues P.C. (2023). Robust local bootstrap for weakly stationary time series in the presence of additive outliers. Stoch. Environ. Res. Risk Assess..

[31] da Silva K.L.S., López-Gonzales J.L., Turpo-Chaparro J.E., Tocto-Cano E., Rodrigues P.C. (2023). Spatio-temporal visualization and forecasting of PM 10 in the Brazilian state of Minas Gerais. Sci. Rep..

[32] Aubrecht C., Fuchs S., Neuhold C. (2013). Spatio-temporal aspects and dimensions in integrated disaster risk management. Nat. Hazards.

[33] Awe O.O., Mahmoudvand R., Rodrigues P.C. (2020). Non-negative time series reconstruction via singular spectrum analysis: A case study of precipitation dynamics in Nigeria. Fluct. Noise Lett..

[34] Oliveira Filho F.M., Guedes E.F., Rodrigues P.C. (2023). Networks analysis of Brazilian climate data based on the DCCA cross-correlation coefficient. PLoS ONE.

[35] Laurini M.P. (2019). A spatio-temporal approach to estimate patterns of climate change. Environmetrics.

